# Resveratrol Inhibits the Invasion of Glioblastoma-Initiating Cells via Down-Regulation of the PI3K/Akt/NF-κB Signaling Pathway

**DOI:** 10.3390/nu7064383

**Published:** 2015-06-02

**Authors:** Yuming Jiao, Hao Li, Yaodong Liu, Anchen Guo, Xiaoxue Xu, Xianjun Qu, Shuo Wang, Jizong Zhao, Ye Li, Yong Cao

**Affiliations:** 1 Department of Neurosurgery, Beijing Tiantan Hospital, Capital Medical University, Beijing 100050, China; E-Mails: shengxiongyuming@163.com (Y.J.); handanlihao-1@163.com (H.L.); liuyaodong666@sina.com (Y.L.); captain9858@vip.sina.com (S.W.); zhaojz205@hotmail.com (J.Z.); 2 China National Clinical Research Center for Neurological Diseases, Beijing 100050, China; E-Mail: guoanchen@hotmail.com; 3 Laborotary of Clinical Medicine Research, Beijing Tiantan Hospital, Capital Medical University, Beijing 100050, China; 4 Medical Experiments and Testing Center, Capital Medical University, Beijing 100069, China; E-Mail: tachee@sina.com; 5 Department of Pharmacology, School of Chemical Biology & Pharmaceutical Sciences, Capital Medical University, Beijing 100069, China; E-Mail: quxj@ccmu.edu.cn

**Keywords:** glioblastoma-initiating cells, RES, invasion, PI3K/Akt, NF-κB, MMP-2

## Abstract

Invasion and metastasis of glioblastoma-initiating cells (GICs) are thought to be responsible for the progression and recurrence of glioblastoma multiforme (GBM). A safe drug that can be applied during the rest period of temozolomide (TMZ) maintenance cycles would greatly improve the prognosis of GBM patients by inhibiting GIC invasion. Resveratrol (RES) is a natural compound that exhibits anti-invasion properties in multiple tumor cell lines. The current study aimed to evaluate whether RES can inhibit GIC invasion *in vitro* and *in vivo*. GICs were identified using CD133 and Nestin immunofluorescence staining and tumorigenesis in non-obese diabetic severe combined immunodeficient (NOD/SCID) mice. Invasive behaviors, including the adhesion, invasion and migration of GICs, were determined by tumor invasive assays *in vitro* and *in vivo*. The activity of matrix metalloproteinases (MMPs) was measured by the gelatin zymography assay. Western blotting analysis and immunofluorescence staining were used to determine the expression of signaling effectors in GICs. We demonstrated that RES suppressed the adhesion, invasion and migration of GICs *in vitro* and *in vivo*. Moreover, we proved that RES inhibited the invasion of GICs via the inhibition of PI3K/Akt/NF-κB signal transduction and the subsequent suppression of MMP-2 expression.

## 1. Introduction

Glioblastoma multiforme (GBM) is one of the most common and fatal tumors originating in the central nervous system, with a median survival time of little more than one year [1]. GBM is characterized by indistinct boundaries and diffuse infiltration throughout the brain parenchyma, which result in unsatisfactory outcomes of surgical excision and/or whole- or partial-brain irradiation [2]. Temozolomide (TMZ) is currently the best chemotherapeutic drug available on the market against GBM and was shown to significantly improve survival in patients with newly diagnosed GBM when administered concomitantly with radiotherapy and as maintenance therapy thereafter [3]. In maintenance therapy, TMZ is usually given in cycles (28 days each). Chemotherapy is administered on the mornings of days 1–5 (treatment period), and no drug is taken on days 6–28 (rest period). The rest period gives normal cells time to recover from the drug’s side effects, and for as long as 23 days, TMZ cannot be administered, despite the risk of invasion and progression of the remaining tumor cells [4].

GBM is composed of heterogeneous cell populations concerning their morphological and differentiation status. It was proposed that a small population of tumor cells named glioblastoma-initiating cells (GICs) play a crucial role in the origin and growth of GBM [5]. After surgical treatment, the residual GICs are thought to be a main cause of tumor recurrence [6,7]. Recent findings have noted that after long-term exposure to therapeutic concentrations of TMZ, differentiated GBM cells convert into glioma stem-like cells and cause the significant expansion of a newly converted GIC population *in vitro* and *in vivo* [8]. Meanwhile, GICs display greater migratory and invasive potential compared with differentiated tumor cells [9,10,11]. The invasion potential of the remaining GICs makes it difficult to prevent the recurrence of GBM, especially during the rest period without TMZ treatment.

Resveratrol (RES) is a polyphenolic antioxidant found in peanuts, grapes and red wine, and although parent RES bioavailability might be insufficient to elicit systemic levels commensurate with cancer chemopreventive efficacy, the anti-oncogenic properties of RES in cells *in vitro* and in rodent models have been amply documented [12,13,14]. In addition, the pharmacologic properties of RES conjugates are unknown, and conjugated metabolites of naturally occurring flavonoids, polyphenols chemically resembling RES, have been suggested to be responsible for, or contribute to, the pharmacologic activity of the parent molecule. Recent studies have elucidated the large role of RES in inhibiting tumor cell adhesion, invasion, and migration by decreasing the invasive phenotype of cancer cells, which alters matrix metalloproteinase (MMP) expression [15]. Previous studies have proven that increased expression of MMPs is involved in tumor invasion and metastasis in many cancer types [16]. To date, over 20 human MMPs have been identified. Among them, MMP-2 and MMP-9 are the enzymes that are most crucial to tumor invasion due to their ability to degrade the extracellular matrix (ECM) and basement membrane [17]. Several upstream pathways controlling MMPs, such as the PI3K/Akt, MAPK, JAK/STAT, and NF-κB pathways, have been implicated in the regulation of invasion by RES [15]. In this study, we investigated the effect of RES against GIC invasion *in vitro* and *in vivo* and studied the underlying mechanisms of RES against highly invasive GICs.

## 2. Experimental Section

### 2.1. Culture of GICs

GICs (400, 411, 412) were derived from neurosurgical samples of three different GBM patients at the Department of Neurosurgery, Beijing Tiantan Hospital, which is affiliated with Capital Medical University. Informed consent was obtained from patients, and the study protocol was approved by the local Ethics Committee. Tumor tissues were washed with Dulbecco’s modified Eagle’s medium (DMEM)/F-12 medium (Invitrogen, Carlsbad, CA, USA) and minced into 1-mm^3^ fragments using scissors. The fragments were then dissociated into single cells by trypsinization. The cells were resuspended and maintained in DMEM/F-12 complete medium consisting of DMEM/F-12 medium, 2 mM GlutaMAX (Invitrogen, Carlsbad, CA, USA), 20 ng·mL^−1^ recombinant human epidermal growth factor (EGF) (R & D, USA), 20 ng·mL^−1^ basic fibroblast growth factor (bFGF) (Invitrogen, USA), an N2 supplement (Invitrogen, USA), and a B27 supplement (Invitrogen, USA). To induce differentiation, the GICs were cultured in DMEM/F12 medium containing 10% fetal bovine serum (FBS) for two weeks.

### 2.2. Determination of GICs in Vitro

To determine the characteristics of GICs *in vitro*, immunofluorescence was performed. Cells grown in suspension or slides were fixed with 4% formaldehyde, permeabilized with 0.1% Triton X-100 for 10 min, and then blocked with 10% normal goat serum (Jackson ImmunoResearch, West Grove, PA, USA) for 5 min. The levels of proteins in the GICs were determined by staining with various primary antibodies at the appropriate dilutions. The primary antibodies included those specific for CD133 (Merck Millipore, Billerica, MA, USA, MAB4399), Nestin (Abcam, Cambridge, UK, ab82375), glial acidic fibrillary protein (GFAP) (GeneTex, inc. Hsinchu city, Taiwan, GTX84438), NF (Cell Signaling Technology, Beverly, MA, USA, #2837), and CNP (Cell Signaling Technology, Beverly, MA, USA, #5664). The cells were incubated at 4 °C overnight. After washing with PBS, the cells were incubated with 100 μL of fluorescein isothiocyanate (FITC)-conjugated secondary antibody (Life Science Technologies, Grand Island, NY, USA). The cell nuclei were stained by 4′,6-diamidino-2-phenylindole (DAPI, Sigma-Aldrich, St Louis, MO, USA) and visualized using a Leica DMI 4000 fluorescent microscope （Leica Microsystems, Wetzlar, Germany).

### 2.3. Determination of GICs in NOD/SCID Mice

The GICs (2 × 10^5^/mice, 6 mice) were injected stereotactically into the right corpus striatum (2.5 mm anterior and 2.5 mm lateral to the bregma and 3.0 mm deep) of six-week-old male NOD/SCID mice (VitalStar, Beijing, China), which were housed in a sterile environment (in a specific pathogen-free room) with a light/dark cycle of 12/12 h. Once the mice were anesthetized, the brains were harvested, fixed with formalin, and then embedded with paraffin. The brains were stained with hematoxylin/eosin (H & E), Nestin (GeneTex, inc. Hsinchu city, Taiwan, GTX39578), and glial acidic fibrillary protein (GFAP) (GeneTex, inc. Hsinchu city, Taiwan, GTX84438). All of the studies involving animals were performed in accordance with the National Institutes of Health Guide for the Care and Use of Laboratory Animals and approved by the local Institutional Animal Care and Use Committee at Beijing Tiantan Hospital.

### 2.4. Cell Viability and Stemness Assay

Cell growth inhibition was determined using a colorimetic assay with MTT followed by RES treatment. Briefly, GICs (400, 411, 412) were seeded in 24-well plates at a density of 2 × 10^4^/well and then treated with RES at various concentrations (0 μM, 5 μM, 10 μM, 20 μM). After 48 h of incubation, cells were incubated with a 3-[4,5-dimethylthiazol-2-yl]-2,5-dipheyl-tetrazolium bromide (MTT, Sigma-Aldrich, USA) solution for another 4 h and then harvested by centrifugation. MTT was solubilized by adding 300 μL of DMSO. The absorbance in each well at 492 nm was measured using a Multiskan Plate Reader (Thermo Scientific, Waltham, MA, USA). To further determine the stemness of GICs, the representative stemness and differentiation markers of GICs (CD133, Nestin and GFAP) were investigated by western blotting analysis.

### 2.5. Cell Adhesion Assay

Ninety-six-well plates were coated with growth factor reduced matrigel (1:300 dilution) overnight at 4 °C. The plates were washed twice with phosphate-buffered saline (PBS) (pH 7.6) and blocked with a solution of 1% BSA/PBS (pH 7.6) for 1 h at 37 °C. GIC cells (5 × 10^4^ cells/well) pre-treated with the indicated concentrations of RES for 48 h were added to the plates and allowed to attach for 30 min at 37 °C. Unattached cells were aspirated, and the cells that were still adherent were stained and fixed with 4% paraformaldehyde and stained with DAPI. The number of adherent cells were counted with a Leica DMI 4000 fluorescence microscope. Each experiment was performed in triplicate and repeated at least three times.

### 2.6. Invasion and Migration Assays

The invasion and migration assays were performed using transwell inserts (Merck Millipore, Billerica, Massachusetts， USA; 8 μm pore size) in 24-well plates. Approximately 5 × 10^4^ cells in 200 μL of serum-free DMEM-F12 medium were placed in the upper chamber, and 600 μL of the GICs medium containing 10% FBS were placed in the lower chamber. For the invasion assay, transwell membranes were precoated with matrigel (BD Biosciences, Bedford, MA, USA). The cells were incubated for 48 h at 37 °C in 5% CO_2_. For the migration assay, the cells were incubated in the uncoated transwells for 24 h. Then, the cells were fixed in 4% paraformaldehyde for 15 min and stained with 0.05% crystal violet in PBS for 30 min. The cells on the upper side of the filters were removed with cotton-tipped swabs, and the filters were washed with PBS. The cells on the underside of the filters were examined and counted with a total 200× magnification under a Leica DMI 4000 microscope. Each experiment was performed in triplicate and repeated at least three times.

### 2.7. Immunofluorescence of NF-κB p65

To determine the translocation of NF-κB p65, immunofluorescence was performed. Cells were seeded in 24-well dishes and incubated with vehicle and 20 μM RES, respectively, for 48 h. Following immunofluorescence, steps were performed as described in “Determination of GICs *in vitro*” except that the primary antibody was anti-NF-κB p65 antibody (Cell Signaling Technology, USA).

### 2.8. Gelatin Zymography Assay

Cells were incubated in serum-free medium for 48 h with different concentrations of RES treatment. The supernatants containing 20 μg of total protein were separated at 4 °C in a 7.5% SDS polyacrylamide gel containing 0.2% gelatin. After electrophoresis, the gels were washed in renaturing buffer (pH 7.5, 2.5% Triton X-100) for 30 min, 4 times; equilibrated in developing buffer (50 mM Tris-HCl pH7.5, 10 mM CaCl_2_, and 1 mM ZnCl_2_) for 30 min; and finally incubated in fresh developing buffer at 37 °C for 24 h to allow digestion of the gelatin. The gel was incubated with staining buffer (0.5% Coomassie blue R-250 in 45% methanol, 10% acetic acid) and then destained with washing buffer (45% methanol, 10% acetic acid) until clear bands suggestive of gelatin digestion appeared. The gel was photographed and then quantitatively measured by scanning densitometry.

### 2.9. Isolation of Nuclear and Cytoplasmic Fractionation

Nuclear and cytoplasmic extracts were prepared with a NE-PER Nuclear and Cytoplasmic Extraction Kit (Thermo Scientific, USA). The purity of nuclear and cytoplasmic extracts was assessed by western blotting with primary antibodies against lamin A (Cell Signaling Technology, USA) and β-actin (Santa Cruz Biotechnology, CA, USA), respectively.

### 2.10. Western Blotting Analysis

GICs (3 × 10^5^/well) seeded in six-well plates and were treated as mentioned previously. The cells were lysed in buffer containing 50 mM HEPES (pH 7.4), 150 mM NaCl, 0.1% Triton X-100, 1.5 mM MgCl_2_, 1 mM EDTA, 2 mM sodium orthovanadate, 4 mM sodium pyrophosphate, 100 mM NaF, and 1:500 protease inhibitor mixture (Roche, Mannheim, Germany). Equal amounts of proteins were separated by 7%–15% SDS-PAGE and then electro-transferred onto a PVDF membrane. The membrane was then blocked with 5% nonfat dry milk for 1 h at room temperature, and the protein levels were determined by overnight incubation with primary antibodies, which included antibodies specific for phospho-IKKα/β (H-470), Akt, phospho-Akt (Ser 473), IKKα/β, JNK, phospho-JNK (Thr183/185), IκBα, phospho-IκBα (Ser 32/36), p38, phospho-p38 (Thr 180), mmp-2, GFAP (Santa Cruz Biotechnology, USA), ERK1/2, phospho-ERK1/2 (Thr202/204), mTOR, phospho-mTOR (Ser 2448), NF-κB p65, Lamin A (Cell Signaling Technology, USA), Nestin (Abcam, Cambridge, UK), CD133 (Miltenyi Biotec, Bergisch Gladbach, Germany) and β-actin. The PVDF membranes were washed in 0.05% Tween-20/TBS and then incubated with a horseradish peroxidase-conjugated secondary antibody. The bound antibodies were visualized using an enhanced chemiluminescent reagent (Merck Millipore, Billerica, Massachusetts， USA) and quantified by densitometry using a ChemiDoc XRS+ image analyzer (Bio-Rad, Hercules, CA, USA). The protein bands were quantified using the ImageJ software (National Institutes of Health, Bethesda, MD, USA), and the results were normalized to the β-actin level.

### 2.11. In Vivo Tumor Invasion Assay

The GICs (2 × 10^5^/mice) were injected stereotactically into the right corpus striatum of six-week-old male NOD/SCID mice as previously described. After 7 days, the tumor-bearing mice were randomly separated into two groups (*n* = 6/group). Mice in group A were treated with intraperitoneal (i.p.) injection of propyleneglycol (vehicle, 0.1 mL), whereas mice in groups B were i.p. injected with 10 mg·kg^−1^ RES (in 0.1 mL propylene glycol) once daily. All of the mice were sacrificed at day 28, and the brains were removed and processed for paraffin embedding. Tumors in the hematoxylin and eosin (H & E) coronal sections were measured to determine the tumor depth of invasion. Slides were photographed with an Aperio CS scanscope (Aperio Technologies, CA, USA) and analyzed at 40× magnification by the ImageScope_v12.0.1.5027 software to assess the depth of invasion. A line was drawn along each evaluable surface, establishing a border of the tumor core without any invading cells or projections of groups of cells. Then, at an interval of 200 to 400 μm along this line, measurements were taken from the previously drawn line to the furthest invading cell perpendicular to the solid line away from the tumor border corresponding to the drawn line. Two independent experiments were performed. The number of measurements per animal averaged 64. The total number of measurements for each tumor was analyzed for statistical significance.

### 2.12. Statistical Analysis

Data were presented as the means ± SD and were analyzed by Student’s *t*-test or one-way ANOVA. Multiple between-group comparisons were performed using the Student-Newman-Keuls (S-N-K) method. A *p*-value <0.05 was considered to be statistically significant. Statistical analysis was performed using the SPSS/Win13.0 software (SPSS, Inc., Chicago, IL, USA).

## 3. Results

### 3.1. Characterization of GICs in Vitro and in Vivo

**Figure 1:**
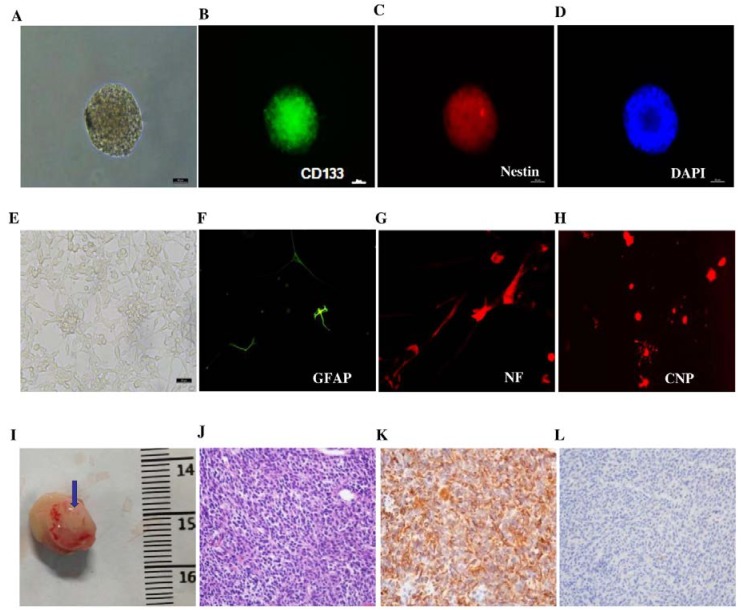
Determination of GICs (412) *in vitro* and *in vivo*. (**A**) Suspended cells formed the self-renewing spheres in stem cell conditioned culture medium (100×); (**B**) Immunofluorescent staining of single suspended cells showed CD133-positive staining (200×); (**C**) Immunofluorescent staining of single suspended cells showed Nestin-positive staining (200×); (**D**) Immunofluorescent staining of DAPI (200×); (**E**) Morphology of serum-induced differentiation of GICs under a microscope (100×); (**F**) Immunofluorescent staining in differentiated GICs showed GFAP-positive staining (200×); (**G**) Immunofluorescent staining in differentiated GICs showed NF-positive staining (200×); (**H**) Immunofluorescent staining in differentiated GICs showed CNP-positive staining (200×); (**I**) The representative image of GIC tumorigenesis in NOD/SCID mice; the tumor is indicated by the blue arrow; (**J**) H & E staining showed the brain xenograft as GBM (200×); (**K**) Immunohistochemical staining in the xenograft section presented high expression levels of Nestin (200×); (**L**) Immunohistochemical staining of xenograft samples presented acicular expression of GFAP (200×).

We sorted CD133^+^ tumor cells from three different surgical specimens of different patients. As shown in Figure 1A–D, Immunofluorescent staining of 412 revealed the presence of stem-cell-related stemness markers such as CD133 and Nestin. Furthermore, as shown in Figure 1E–H, CD133^+^ cells also exhibited multilineage differentiation potential, demonstrated by high expression levels of GFAP (astrocytes)-, NF (neuron)-, and CNP (oligodendrocytes)-directed morphology in accordance of differentiation. The characteristics of GICs were also confirmed by the assay of tumorigenesis in NOD/SCID mice. As shown in Figure 1I, GICs have the distinguished character of tumorigenesis. The brain tumors were determined through H & E staining (Figure 1J). The brain samples also presented high expression levels of Nestin (Figure 1K) and acicular expression of GFAP (Figure 1L). The other two cell lines showed similar characteristics.

### 3.2. Effects of RES on GIC Viability and Stemness

**Figure 2:**
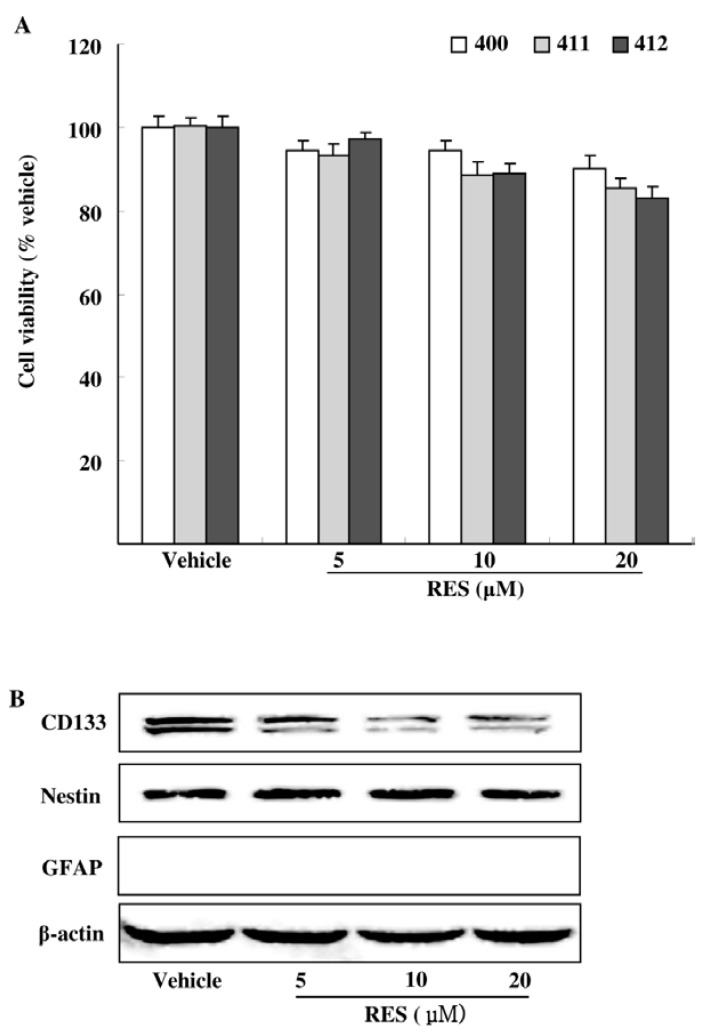
Effects of RES on the viability and stemness of GICs. (**A**) GICs (400, 411, 412) were treated with dimethyl sulfoxide (DMSO, vehicle) and 5 μM, 10 μM, and 20 μM RES for 48 h, respectively. Cell viability was examined by the MTT assay; (**B**) Representative GICs (412) were treated with DMSO (vehicle) and 5 μM, 10 μM, and 20 μM RES for 48 h, respectively. CD133, Nestin and GFAP were investigated by Western blotting analysis. PC-12 lysate was used as the positive protein of GFAP (results shown in Supplementary Data).

We first examined the cytotoxicity of RES at the concentration below 20 μM on GICs (400, 411, 412). By the MTT assay, we found that RES had mild cytotoxicity to GICs. The administrations of RES 20 μM for 48 h reduced GICs by 9.76%, 14.58% and 16.99%, respectively. (Figure 2A). To further determine the stemness of GICs, the representative stemness and differentiation markers of GICs (CD133, Nestin and GFAP) were investigated by Western blotting analysis. We noticed a decrease of CD133 but without the decrease of Nestin and the appearance of differentiation marker (GFAP) (Figure 2B). These results suggested that RES at the concentration below 20 μM exhibited low toxicity and had little effect on the survival and stemness of GICs, but showed a specific inhibition of CD133.

### 3.3. RES Inhibits Adhesion, Migration, and Invasion of GIC Cells in Vitro

**Figure 3:**
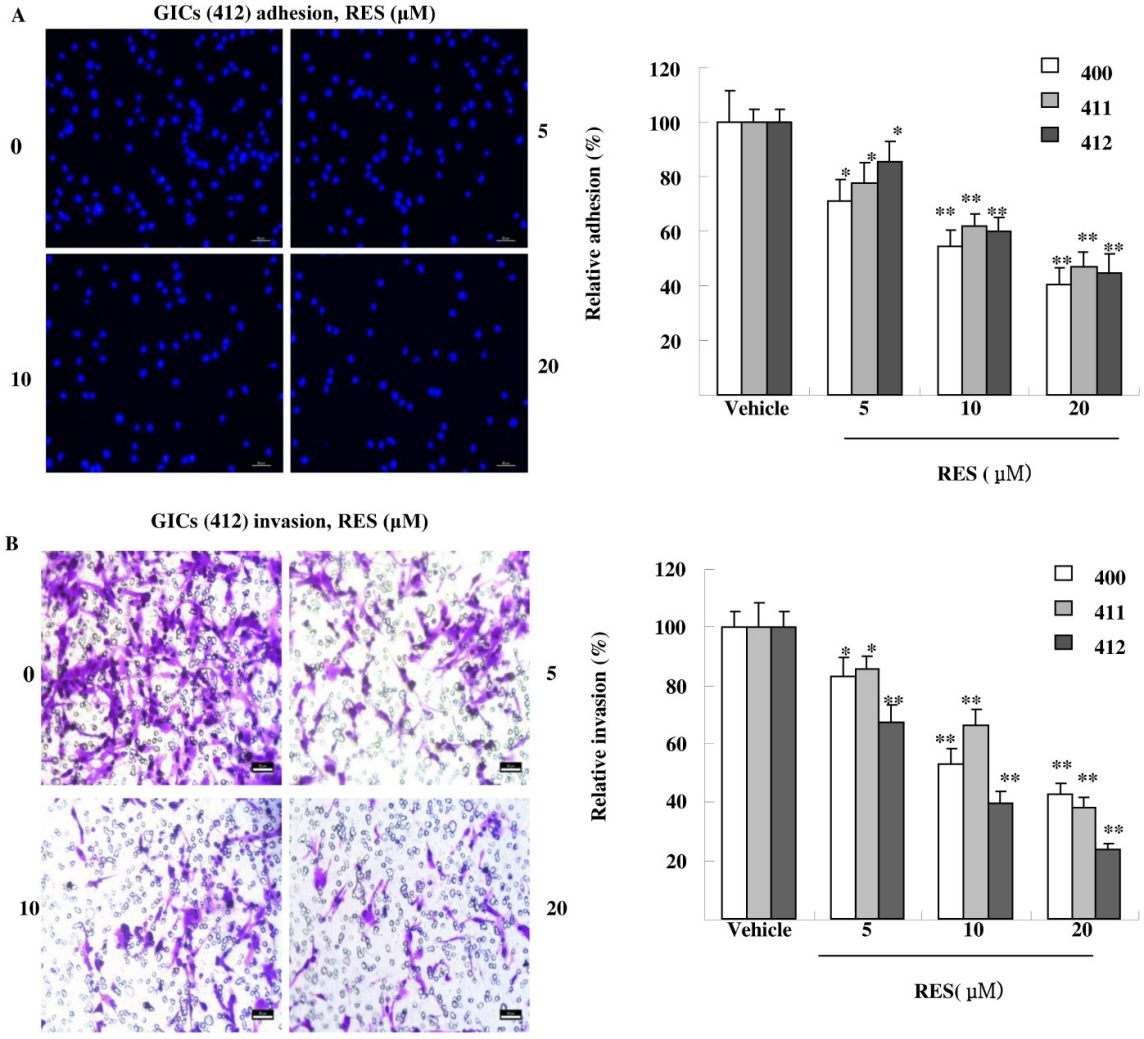

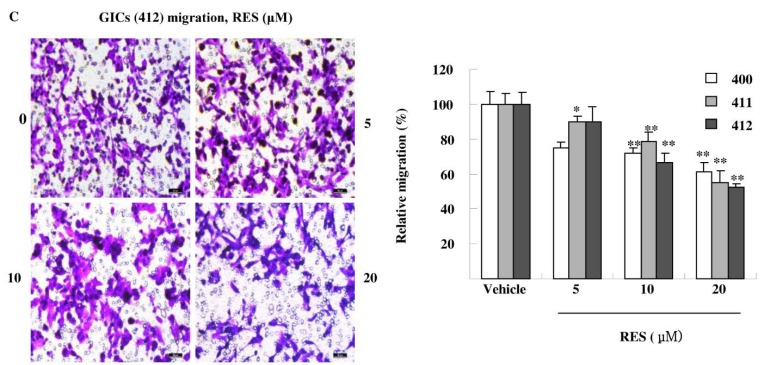
RES reduces the adhesion, invasion and migration of GICs. (**A**) GICs (400, 411, 412) were seeded in a 96-well plate coated with matrigel and treated with DMSO (vehicle) and 5 μM, 10 μM, and 20 μM RES for 48 h, respectively. Attached cells were photographed (200×) after DAPI staining, and the number of attached cells was calculated as a percentage of adhesion. Data are shown as the means ± SD of three independent experiments by Student’s *t*-test. * *p* < 0.05, ** *p* < 0.01, *vs.* Vehicle; (**B**) GICs (400, 411, 412) were seeded in transwells pre-coated with matrigel and treated with DMSO (vehicle) and 5 μM, 10 μM, and 20 μM RES for 48 h, respectively. Then, randomly chosen fields were photographed (200×), and the number of cells migrated to the lower surface was calculated as a percentage of invasion. Data are shown as the mean ± SD of three independent experiments by Student’s *t*-test. * *p* < 0.05, ** *p* < 0.01, *vs.* Vehicle; (**C**) GICs (400, 411, 412) were seeded in transwells without matrigel and were treated with DMSO (vehicle) and 5 μM, 10 μM, and 20 μM RES for 48 h, respectively. Then, randomly chosen fields were photographed (200×), and the number of cells that migrated to the lower surface was calculated as a percentage of migration. Data are shown as the means ± SD of three independent experiments by Student’s *t*-test. * *p* < 0.05, ** *p* < 0.01, *vs.* Vehicle.

Tumor cells exhibit a variety of properties, including altered adhesiveness, increased motility and invasive capacity, to complete the invasion process [18]. As shown in Figure 3A, RES significantly decreased GICs adhesion in a dose-dependent manner. RES treatment (20 μM) suppressed GICs (400, 411, 412) adhesion to matrigel by 45.57%, 52.86% and 55.34%, respectively. As shown in Figure 3B, RES also inhibited GICs invasion in a concentration-dependent manner. RES treatment (20 μM) reduced the invasion ability of GICs by 57.54.3%, 61.84% and 76.30%, respectively. Finally, the effect of RES on cell migration was detected. As shown in Figure 3C, the migrated cells treated with 20 μM RES went through the transwell membrane were decreased by 38.49%, 45.21%, and 47.43%, respectively. These results demonstrate that treatment with RES dramatically attenuated invasion of GICs at non-cytotoxic concentrations in a concentration-dependent manner.

### 3.4. RES Suppresses the Activity and Expression of MMP-2 in GICs

**Figure 4:**
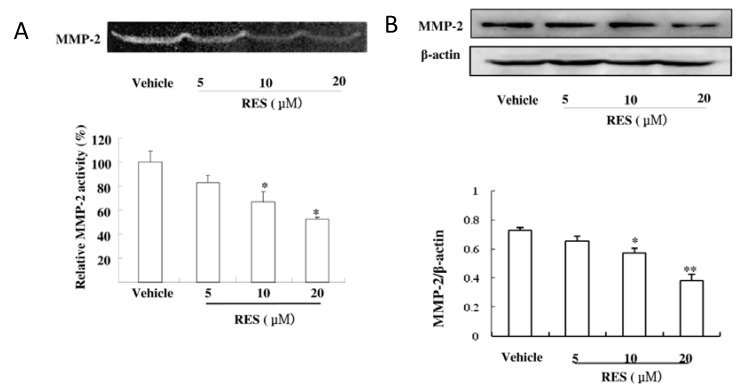
RES suppresses the activity and expression of MMP-2 in GICs. (**A**) GICs (412) were treated with DMSO (vehicle) and 5 μM, 10 μM, and 20 μM RES for 48 h, respectively. The activity of MMP-2 was assessed using the concentrated conditioned medium (CM) by gelatin zymography; (**B**) Cell lysates were subjected to western blotting with anti-MMP-2 antibody to evaluate the protein expression of MMP-2. β-actin was used as a loading control. * *p* < 0.05, ** *p* < 0.01, *vs.* Vehicle.

We further determined the activity and expression of MMP-9 and MMP-2 by applying gelatin zymography and western blotting. As shown in Figure 4A, RES markedly reduced the gelatinolytic activity of MMP-2 produced from GICs cells; however, low activity of MMP-9 was observed in GICs. Consistent with the decrease of gelatinolytic activity, western blotting analysis also revealed that RES down-regulated the level of MMP-2 expression (Figure 4B). These results indicated that RES inhibited the invasiveness of GICs cells by decreasing both the activity and expression of MMP-2.

### 3.5. RES Represses Cell Invasion by Inhibiting the Activation of the NF-κB Pathway

**Figure 5:**
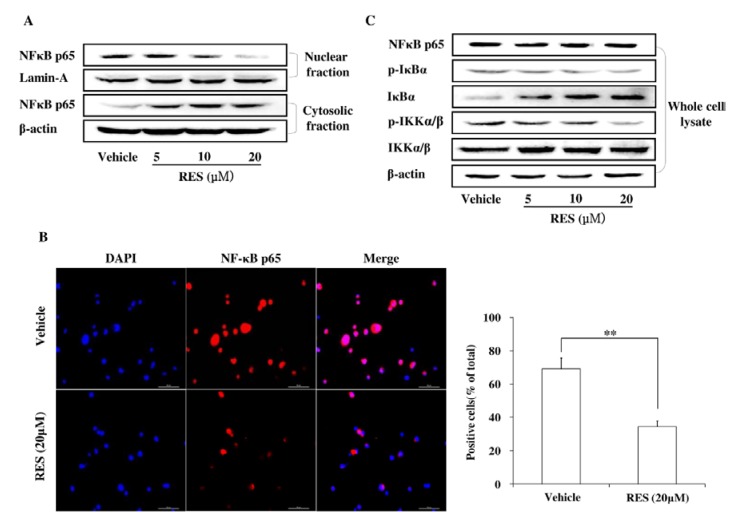
RES suppresses GIC invasion through the inhibition of the NF-κB pathway. (**A**) GICs (412) were treated with DMSO (vehicle) and 5 μM, 10 μM, and 20 μM RES for 48 h, respectively. Cytoplasmic and nuclear fractions of GICs were isolated, and the concentration of nuclear and cytoplasmic protein was measured by western blotting with an anti-NF- kB p65 antibody. Lamin A and β-actin were used as loading controls; (**B**) GICs (412) were treated with DMSO (vehicle) or 20 μM RES for 48 h and then immunostained with anti-NF-κB p65 and DAPI. Then, randomly chosen fields were photographed (400×) and the number of positive cells was calculated. Data are shown as the mean ± SD of three independent experiments by Student’s *t*-test. * *p* < 0.05, ** *p* < 0.01, *vs.* Vehicle; (**C**) GICs (412) were treated with DMSO (vehicle) and 5 μM, 10 μM, and 20 μM RES for 48 h, and the expression of target proteins was detected by western blotting using anti-NF-κB P65, anti-IκBα, anti-p-IκBα, anti-p-IKKα/β, anti-IKKα/β antibodies. β-actin was used as a loading control.

The NF-κB pathway was investigated by western blotting analysis. The data in Figure 5A demonstrated that RES inhibited the nuclear translocation of NF-κB p65, with a decreased nuclear level but an increased cytoplasmic level, and this effect was confirmed by immunofluorescence assay (Figure 5B). Meanwhile, the phosphorylation levels of IKKα/β and IkBα were detected by western blotting. As shown in Figure 5C, RES efficiently inhibited the phosphorylation of IKKα/β and IkBα, whereas the expression of IKKα/β remained unchanged. These results further indicated that RES suppressed cell invasion by inhibiting NF-κB activation, which is the upstream activator for MMP-2 expression.

**Figure 6:**
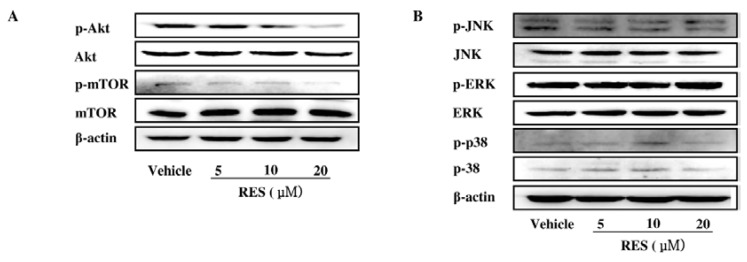

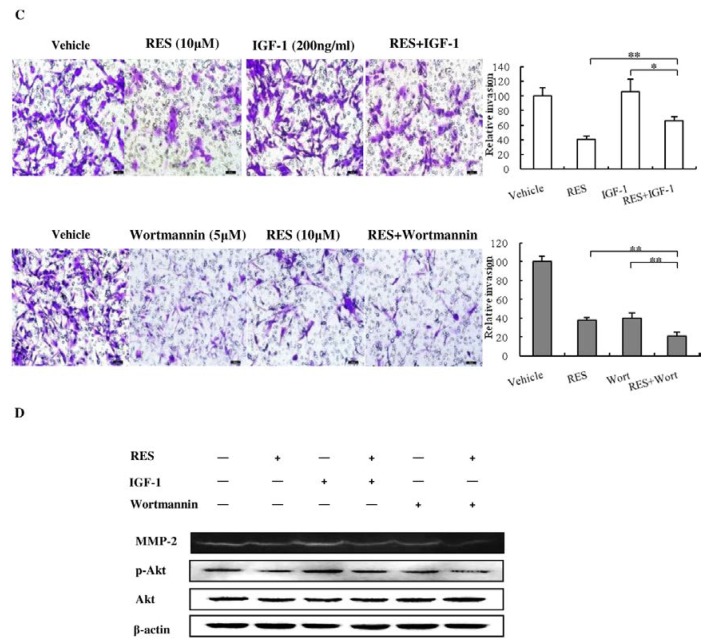
PI3K/AKT signaling pathway is involved in the inhibitory effect of RES on NF-κB activation. (**A**) GICs (412) were treated with DMSO (vehicle) and 5 μM, 10 μM, and 20 μM RES for 48 h. Total expression and phosphorylation of Akt and mTOR were analyzed by western blotting using anti-Akt, anti-p-Akt, anti-mTOR, anti-p-mTOR, antibodies. β-actin was used as a loading control; (**B**) Total expression and phosphorylation of JNK, ERK, and p38 were analyzed by western blotting using anti-JNK, anti-p-JNK, anti-ERK, anti-p-ERK, anti-p38 anti-p-p38 antibodies. β-actin was used as a loading control; (**C**) GICs (412) were pre-treated with IGF-1 (200 ng·mL^−1^) or wortmannin (5 μM) for 30 min and then incubated in the presence or absence of RES (10 μM). GICs were then subjected to cell invasion assay. Then, randomly chosen fields were photographed (200×), and the number of cells that migrated to the lower surface was calculated as a percentage of invasion. Data are shown as the mean ± SD of three independent experiments by Student’s *t*-test. * *p* < 0.05, ** *p* < 0.01; (**D**) GICs were pre-treated with IGF-1 (200 ng·mL^−1^) or wormannin (5 μM) for 30 min and incubated in the presence or absence of RES (10 μM) for 48 h, and then, the cell lysates were subjected to western blotting with anti-p-Akt and anti-Akt antibodies. β-actin was used as a loading control. The CM were subjected to gelatin zymography assay to determine the activity of MMP-2.

### 3.6. The PI3K/AKT Signaling Pathway is Involved in the Inhibitory Effect of RES on NF-κB Activation

It was reported that the MAPK and PI3K/AKT pathways can regulate fundamental cellular processes by the induction of IKK-dependent NF-κB activation [19,20]. In our study, RES significantly reduced the phosphorylation of Akt and mTOR in GICs without affecting the total level (Figure 6A). However, RES had little effect on the MAPK signaling pathway, which was supported by the unchanged phosphorylation of the ERK1/2, p38, and JNK pathways (Figure 6B).

To confirm whether the suppression of the invasion of GICs by RES was mainly regulated through the PI3K/Akt pathway, insulin-like growth factor-1 (IGF-1), one of the most potent activators of PI3K/Akt signaling pathway [21], and wortmannin (a strong PI3K/Akt inhibitor [22]) were used to evaluate the underlying mechanism. As shown in Figure 6C and Figure 6D, treatment with RES (10 μM) substantially inhibited the phosphorylation of Akt induced by IGF-1 as well as the IGF-1-activated secretion of MMP-2 and subsequent cell invasion. Meanwhile, wortmannin markedly decreased the levels of the phosphorylated Akt as well as the secretion of MMP-2 and subsequent cell invasion. In addition, RES enhanced the effects of wortmannin on the phosphorylation of Akt, the secretion of MMP-2, and GIC invasion. Taken together, these results indicate that RES could suppress GIC invasion by inhibiting the PI3K/Akt pathway.

### 3.7. RES Reduces Invasion of GICs in Vivo

**Figure 7:**
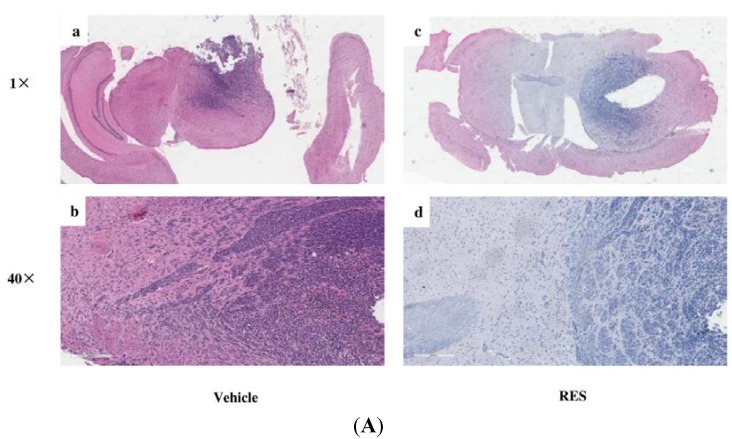

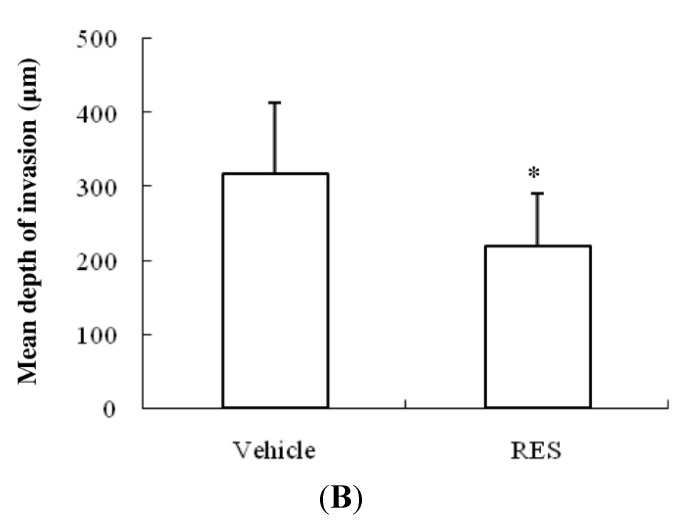
RES reduces invasion of GICs *in vivo*. 2 × 10^5^ GICs (412) in a volume of 5 μL PBS were stereotactically implanted into the brains of mice. Then, the mice were treated with propylene glycol (vehicle, 0.1 mL) or 10 mg·kg^−1^ RES (in 0.1 mL propylene glycol), respectively. (**A**) Representative results of H & E staining of GBM-bearing NOD/SCID mouse brain paraffin sections; (**a**) An H & E staining section of the brain from an NOD/SCID mouse treated with vehicle (1× original magnification); (**b**) a representative enlarged regional field (40× magnification) from (a); (**c**) an H & E staining section of the brain from an NOD/SCID mouse treated with RES (1× original magnification); (**d**) a representative enlarged regional field (40× magnification) from (**c**); (**B**) The depth of tumor cell invasion was evaluated as described in the “Materials and Methods” section. The bar graph represents the average depth of invasion (μm) at the edge of tumors derived from mice treated with vehicle or RES (*n* = 6/group). Data are shown as the mean ± SD. * *p* < 0.05, *vs.* Vehicle.

As shown in Figure 7, tumors treated with RES were significantly less invasive compared with control tumors treated with vehicle, as assessed by H & E staining. The control tumors have an irregular shape with single cells and cell clusters that invade along white matter tracts as well as into gray matter (Figure 7A–a,b). In contrast, mice treated with RES formed oval shaped tumors with a smoother border (Figure 7A–c,d). The mean depth of invasion in the control tumors was 317.9 ± 95.6 μm compared with 219.2 ± 70.7 μm in tumors treated with RES (*p* < 0.05, Figure 7B). These results suggested that RES could suppress GIC invasion *in vivo*.

## 4. Discussion

In the current study, we showed that RES conspicuously inhibited the invasive behaviors of GICs *in vitro* and *in vivo* at a rather low concentration. The underlying mechanisms in this process involved the suppression of the PI3K/Akt/NF-κB signaling pathway and inhibition of MMP-2 secretion.

In most previous studies, RES exerts its effect in the middle or high micromolar range. RES at 100 μM induces apoptosis through the activation of caspase-3 in a human glioma U251 cell line [23]. At 50 μM, RES inhibits TNF-α-mediated MMP-9 expression and invasion of human hepatocellular carcinoma cells [24]. However, in the circulation, attainable concentrations of RES are in the low micromolar range [25]. In the current study, we showed that RES, at a relative low dose of 20 μM, effectively inhibited GICs’ invasive behaviors. Tumor invasion is a complex and multistage process. A multistep model of invasion suggests that cancer cells must first adhere to the ECM (adhesion), proteolytically degrade the matrix (invasion), and finally migrate through this barrier to surrounding tissue (migration) [26]. In a previous study, Ryu *et al.* (2011) showed that RES reduces TNF-α-induced U373MG human glioma cell activity in the invasion phase [27]. In our study, we show that RES could effectively restrain the invasive behaviors of GICs in the adhesion, invasion, and migration phases.

MMPs are a family of metal-containing enzymes. Evidence of the important role of MMPs in the invasive potential of tumors *in vitro* and *in vivo* has been well documented [28]. Meanwhile, the overexpression MMPs has been reported in cancer stem cells (CSCs). Yang *et al.* (2011) found that there is an upregulation of MMP expression in gastric cancer stem-like cells compared with gastric cancer tumor cells [29]; increased MMP-2 significantly contributes to their enhanced capabilities of invasion *in vitro* and metastasis *in vivo*. Some studies have proven that RES reduces tumor invasion by suppressing MMPs. Tang *et al.* (2008) showed that RES can inhibit MMP-9 expression and cell invasion in human breast cancer cells [30]. Weng *et al.* (2010) demonstrated that RES down-regulates the expression of MMP-2 and MMP-9 in human hepatocarcinoma cells [31]. Our acquired data manifested that RES could inhibit gelatinolytic activity and suppress the expression of MMP-2 in GICs and that MMP-2 was a RES-responsive mediator, which leads to the degradation of ECM, causing subsequent tumor invasion and migration.

A recent study showed that glioma CSCs present constitutive activation of the NF-κB signaling pathway and the up-regulation of NF-κB-dependent genes [32]. The NF-κB pathway can work as the upstream regulatory elements of the transcription of the MMP-2 gene [33]. Our data indicated that RES inhibited the activation of NF-κB and exerted this effect by blocking IkB-a phosphorylation and NF-κB P65 translocation. It has been widely accepted that the PI3K/Akt or MAPK signaling pathway is an important upstream element for the regulation of NF-κB activation in multiple tumor cell lines. Tang *et al.* (2008) demonstrated that RES inhibits heregulin-β1-mediated MMP expression and cell invasion in human breast cancer cells through regulation of the MAPK/ERK signaling pathway [30]. Sheth *et al.* (2012) showed that RES reduces prostate cancer growth and metastasis by inhibiting the PI3K/Akt pathway [34]. Our data showed that the PI3K/Akt signaling pathway rather than the MAPK signaling pathway was involved in the inhibition of NF-κB activation by RES and that RES could reduce the activation of the PI3K/Akt signal pathway induced by insulin-like growth factor-1 (IGF-1). Therefore, PI3K/Akt might be an effective target for RES in influencing GIC invasion behaviors.

At high or median doses, RES can decrease CSC cell proliferation, leading to apoptosis, and it can induce GIC differentiation [35,36]. Reducing CSCs’ invasive power can be achieved by affecting their viability and stemness. Our study showed that low-dose RES did not inhibit the growth of GICs and that there were no obvious alterations in the expression of Nestin and GFAP in the GICs, which implied that low-dose RES did not alter GICs’ viability and their stemness. However, our result also showed that the expression of another CSC surface marker, CD133, decreased after treatment with RES, which was inconsistent with previous data. CD133 is a transmembrane glycoprotein and is used to isolate and characterize CSCs from GBM specimens. Mechanisms regulating the expression and modification of the CD133 epitope remain unclear; its use as a universal marker to identify GBM stem cells is still controversial [37]. Meanwhile, a recent study indicated that a regulatory relationship exists between CD133 and the PI3K/Akt pathway. As an upstream element, CD133 knockdown potently inhibits the activity of the PI3K/Akt pathway [38]. Whether RES decreases CD133 to inactivate the PI3K/Akt pathway in GIC invasion needs further research.

## 5. Conclusions

In conclusion, our study demonstrates that low-dose RES significantly inhibited GICs’ invasive abilities, which might imply that administrating RES during the rest period of TMZ maintaining therapy may be a potential choice for inhibiting tumor infiltration and metastasis.

## Supplementary Files

Supplementary File 1
