# Supplementary figures and images for: Resveratrol Inhibits the Invasion of Glioblastoma-Initiating Cells via Down-Regulation of the PI3K/Akt/NF-κB Signaling Pathway
Source: Nutrients. 2015 Jun 2;7(6):4383–402. doi: 10.3390/nu7064383 (PMC4488790; doi:10.3390/nu7064383)

**Supplementary Information**


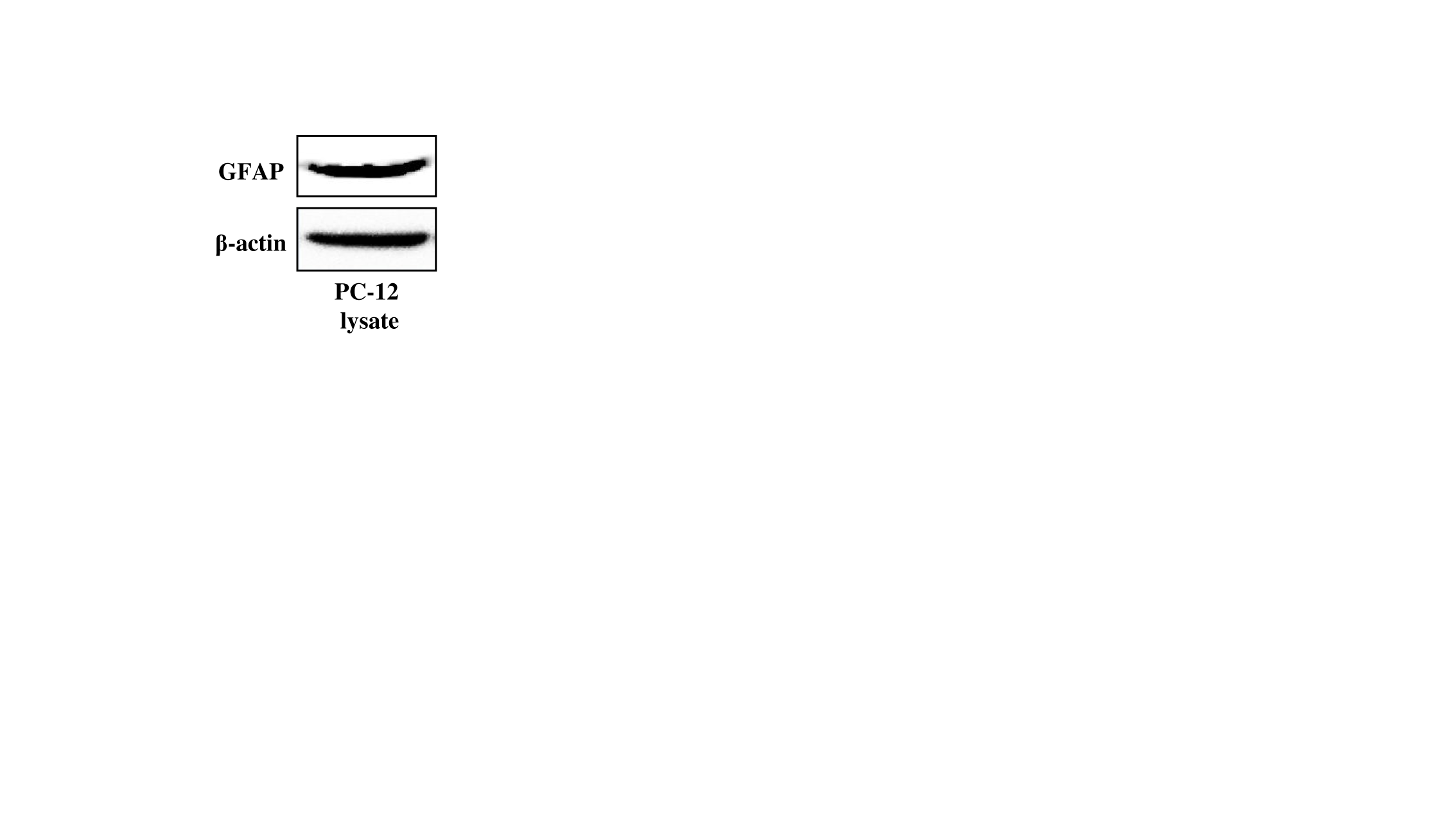


**Figure S1.** PC-12 lysate was used as the positive protein of GFAP.

Supplement: Supplementary File 1 [file nutrients-07-04383-s001.docx]
